# *Burkholderia* Phages and Control of *Burkholderia*-Associated Human, Animal, and Plant Diseases

**DOI:** 10.3390/microorganisms13081873

**Published:** 2025-08-11

**Authors:** Bingjie Wang, Jiayi Zhang, Lei Chen, Munazza Ijaz, Ji’an Bi, Chenhao Li, Daixing Dong, Yanxin Wang, Bin Li, Jinyan Luo, Qianli An

**Affiliations:** 1 State Key Laboratory of Rice Biology and Breeding, Ministry of Agriculture and Rural Affairs Key Laboratory of Molecular Biology of Crop Pathogens and Insect Pests, Zhejiang Key Laboratory of Biology and Ecological Regulation of Crop Pathogens and Insects, Zhejiang Engineering Research Center for Biological Control of Crop Pathogens and Insect Pests, Institute of Biotechnology, Zhejiang University, Hangzhou, 310058, China; 22316264@zju.edu.cn (B.W.); 22316069@zju.edu.cn (J.Z.); drmunazzaijaz@zju.edu.cn (M.I.); libin0571@zju.edu.cn (B.L.); 2 Department of Plant Quarantine, Shanghai Extension and Service Center of Agriculture Technology, Shanghai 201103, China; chenlei200524@163.com; 3 Crop Institute, Ningbo Academy of Agricultural Sciences, Ningbo 315040, China; bihappy@foxmail.com; 4 Station for the Plant Protection & Quarantine and Control of Agrochemicals Zhejiang Province, Hangzhou 310004, China; lichenh2021@zju.edu.cn; 5 Fuyang District Agricultural Technology Extension Center, Hangzhou 311400, China; 15167120905@163.com; 6 Hangzhou Agricultural Technology Extension Center (Hangzhou Plant Protection and Quarantine Center), Hangzhou 310020, China; yanxin5200@126.com

**Keywords:** bacteriophage, *Burkholderia*, bacterial panicle blight, cystic fibrosis, nanocarrier, sustainable disease management

## Abstract

Gram-negative *Burkholderia* bacteria are known for causing diseases in humans, animals, and plants, and high intrinsic resistance to antibiotics. Phage therapy is a promising alternative to control multidrug-resistant bacterial pathogens. Here, we present an overview of *Burkholderia* phage characteristics, host specificity, genomic classification, and therapeutic potentials across medical, veterinary, and agricultural systems. We evaluate the efficacy and limitations of current phage candidates, the biological and environmental barriers of phage applications, and the phage cocktail strategy. We highlight the innovations on the development of targeted phage delivery systems and the transition from the exploration of clinical phage therapy to plant disease management, advocating integrated disease control strategies.

## 1. Introduction

*Burkholderia sensu lato* are Gram-negative bacteria within the family *Burkholderiaceae*, order *Burkholderiales*, and class *Betaproteobacteria*. *Burkholderia sensu lato* has been divided into *Burkholderia sensu stricto* and other six genera named *Paraburkholderia*, *Caballeronia*, *Robbsia*, *Mycetohabitans*, *Trinickia*, and *Pararobbsia* [1]. The genus *Burkholderia sensu stricto* currently comprises 36 validly published species (https://lpsn.dsmz.de/genus/burkholderia) (accessed on 10 May 2025). *Burkholderia sensu stricto* has wide metabolic versatility and adaptation to versatile lifestyles as free-living bacteria in soil or water and as commensals of plants, animals, or fungi [1]. The genus *Burkholderia sensu stricto* is phylogenetically divided into three major species complexes: *Burkholderia cepacia* complex (Bcc), *Burkholderia pseudomallei* complex (Bpc), and *Burkholderia glumae* complex (Bgc). Bcc includes *B. cepacia*, *B. cenocepacia*, *B. ambifaria*, *B. contaminans*, *B. multivorans*, *B. stabilis*, and *B. vietnamiensis*; Bpc includes *B. pseudomallei*, *B. mallei*, *B. thailandensis*, *B. oklahomensis*, and *B. singularis*; and Bgc includes *B. glumae*, *B. gladioli*, and *B. plantarii* [2].

Although some members of the genus *Burkholderia sensu stricto* show biotechnological potentials of plant growth promotion, biocontrol, antibiotic production, biodegradation, and bioremediation, major members are pathogens to human, animals, and plants. Bcc members, such as *B. cenocepacia* and *B. multivorans*, are well-known pathogens that cause chronic pulmonary infections in cystic fibrosis (CF) [3,4]. Bpc members, such as *B. pseudomallei*, are etiological agents of melioidosis, a potentially fatal disease endemic to tropical regions [5]. Bgc members *B. glumae* and *B. gladioli* cause bacterial panicle blight (BPB) in rice, while *B. plantarii* causes rice seedling blight and grain rot [2]. Of particular concern to Bgc is their potential to be opportunistic human pathogens to various immunocompromised populations [6,7,8,9].

The genus *Burkholderia sensu stricto* is characterized by large and complex genomes, typically comprising multiple replicons (chromosomes and plasmids) that encode extensive repertoires of genes for environmental adaptation and metabolic plasticity [10]. The large and complex genomes facilitate environmental adaptation, allowing for the colonization of diverse niches, including soil ecosystems, aquatic environments, plant rhizospheres, and even intracellular compartments of eukaryotic hosts [11]. This genomic architecture also facilitates high levels of antibiotic resistance, posing serious challenges for both clinical treatment and agricultural disease control [11,12]. Of particular concern is the increasing prevalence of multidrug-resistant *Burkholderia* strains in clinical settings where therapeutic options are severely constrained [13]. In agriculture, overreliance on chemical pesticides has further driven resistance and raised environmental hazards. These challenges have spurred interest in alternative approaches, notably phage therapy [14].

Phages, also known as bacteriophages, are viruses that specifically infect and lyse bacteria, offering a targeted biocontrol strategy against *Burkholderia* infections. Phage action involves specific recognition of bacterial surface receptors, the injection of viral genetic material, and hijacking of the host’s cellular machinery for replication. This process culminates in cell lysis, releasing new phages to continue the cycle [15]. Due to the high specificity, phages offer an alternative to conventional antibiotics, particularly for managing multidrug-resistant strains. In clinical settings, phages have shown efficacy against multidrug-resistant strains where antibiotics fail [15,16,17,18,19]. For example, phage C34 targeting *B. pseudomallei* significantly reduced bacterial load and improved survival rates in infected mice [17]. In agriculture, phage NBP4-7 and jumbo phage S13 reduced BPB severity in rice by targeting key virulence factors like flagella [20,21].

Phage therapy presents a promising cross-disciplinary solution for managing *Burkholderia*-induced diseases in humans, animals, and plants. The advance of phage therapy in human and veterinary medicine provides an adaptation strategy for plant disease management. In clinical settings, phages are administered through intravenous, oral, and topical routes with formulations optimized for stability and therapeutic efficacy [22]. In agriculture, phages are typically applied via foliar sprays, seed treatments, or soil drenches depending on the crops and pathogens [23,24]. However, phage application in cropland is vulnerable to environmental inactivation and degradation by high temperature, UV radiation, drought, agrochemicals, and soil absorption. Recent development in delivery technologies, particularly phage encapsulation in nanocarriers, enhances phage viability and site-specific release [25]. These delivery technologies can be translated to protect phages targeting Bgc members in cropland [26].

Here, we review the advances in research on *Burkholderia* phages, particularly on *Burkholderia* phage therapeutic potential across host systems. We highlight the delivery innovations and cross-application strategies that may enhance the integration of phage therapy into sustainable disease management programs.

## 2. Pathogenic *Burkholderia* Species

### 2.1. Human and Animal Pathogens

The genus *Burkholderia sensu stricto* includes pathogenic species that pose significant threats to human and animal health (Table S1). Among these, members of Bcc such as *B. cepacia*, *B. multivorans*, and *B. cenocepacia* are well-known opportunistic pathogens. They are most frequently isolated from individuals with CF and chronic granulomatous disease, where they are associated with severe respiratory infections, including necrotizing pneumonia and septicemia [27,28]. *B. dolosa* and *B. anthina* have been linked to accelerated pulmonary decline and chronic obstructive pulmonary diseases, respectively [29,30]. Beyond respiratory infections, Bcc contributes to bloodstream infections, wound contaminations, and sepsis. *B. stabilis* and *B. contaminans* have been associated with nosocomial infections and bacteremia, posing challenges in hospital settings [31,32,33,34]. *B. pseudomultivorans* was first isolated from clinical CF sputum and rhizosphere soil [35]. Recently, *B. pseudomultivorans* was identified as the cause of sepsis in cats, suggesting zoonotic potential [36].

Within Bpc, *B. pseudomallei* causes melioidosis, a severe zoonotic disease endemic to Southeast Asia and northern Australia. It infects a broad-host range including humans, domestic animals, wildlife, and pets. Clinical presentations include pneumonia, sepsis, abscesses, and chronic infections [37,38]. *B. mallei* causes glanders, a zoonotic disease primarily affecting horses, donkeys, and mules. *B. mallei* was weaponized during World War I due to its high infectivity [39,40,41]. *B. thailandensis* is less virulent and typically causes opportunistic infections in immunocompromised individuals [42,43,44].

Members of the Bcc possess multiple virulence factors (Table 1) including biofilm formation, motility, quorum sensing, pili, LPS variation, secretion systems, and extracellular enzymes that enable colonization and immune evasion. They exhibit intrinsic resistance mechanisms such as efflux pumps, β-lactamases, low membrane permeability, modified LPS, and polymyxin resistance in some species [45,46,47,48,49,50,51]. Similarly, Bpc species like *B. pseudomallei* display virulence traits including biofilm formation, motility, intracellular survival, capsular polysaccharide, quorum sensing, diverse secretion systems (Type III, V, VI), and adhesins [14,52,53,54]. Both groups share resistance features, including multidrug efflux pumps and β-lactam resistance. Effective management typically requires carbapenems or β-lactam/β-lactamase inhibitor combinations. However, persistent infections and relapse are common due to biofilm formation and adaptive resistance [14].

### 2.2. Plant Pathogens

Several Bgc members (*B. glumae*, *B. gladioli*, and *B. plantarii*) and Bcc members (*B. cepacia*, *B. orbicola*, *B. semiarida* and *B. sola*) are recognized as plant pathogens, causing substantial agricultural losses (Table S1). *B. glumae* is a major pathogen responsible for BPB in rice, causing symptoms like aborted seeds, empty grains, and seedling rot, significantly reducing rice yield [55,56]. *B. glumae* also infects other crops such as pepper, eggplant, tomato, sesame, and perilla [50]. Notably, *B. glumae* has also been isolated from human clinical cases, indicating its potential for cross-kingdom pathogenicity [6,57]. Likewise, *B. gladioli* infects rice and a variety of other crops, causing grain rot and seedling blight. *B. gladioli* also acts as an opportunistic human pathogen, causing bacteremia, pneumonia, and lung infections in CF patients [58,59,60,61]. *B. plantarii* primarily infects rice, leading to seedling blight, grain rot, chlorosis, and stunting [62,63]. *B. cepacia* causes bulb rot in onions [64]. *B. orbicola* reduces bean seed germination and impairs insect survival [59]. *B. semiarida* and *B. sola* are associated with onion sour skin disease [65,66]. These pathogens exhibit biofilm formation, motility (flagella), quorum sensing systems, and secretion systems (including type III), and produce toxins like toxoflavin and extracellular enzymes that facilitate host tissue colonization and damage [67,68,69,70,71,72]. They possess resistance to conventional control measures, making them agriculturally significant threats.

**Table 1:** Comparative summary of major pathogenic *Burkholderia*.

Species	Host Range	Key Virulence Factor	Resistance Trait	Zoonotic Risk	Reference
*B. cepacia*	Humans, occasionally animals, plants	Biofilm formation, motility, pili, lipopolysaccharide variation, quorum sensing (QS), extracellular enzymes	Efflux pumps, β-lactamases, low permeability, modified lipopolysaccharide	Opportunistic zoonotic risk	[45,46]
*B. multivorans*	Humans (CF)	Biofilm formation, motility, cable pili, QS-controlled virulence	Aminoglycoside, β-lactam resistance, efflux pumps, polymyxin resistance	No known zoonotic transmission	[14,46]
*B. cenocepacia*	Humans (CF, immunocompromised)	Biofilm formation, motility, QS-regulated proteases, cable pili, secretion systems, siderophore production	Efflux pumps, β-lactamases, polymyxin resistance	Potential zoonotic pathogen	[14,46,47,48]
*B. dolosa*	Humans (CF)	Biofilm and capsule formation, motility, adhesins and proteases, secretion systems	Extensive multidrug resistance, multiple efflux pumps, β-lactamases	No known zoonotic transmission	[49,50]
*B. contaminans*	Humans (nosocomial)	Biofilm formation, motility, hemolysins, antifungal activity, secretion systems	β-lactams, disinfectants, efflux pumps	Potential zoonotic pathogen	[33,46,51]
*B. pseudomallei*	Humans and animals	Biofilm formation, motility, intracellular survival, polysaccharides, QS, secretion systems, immune evasion	Aminoglycosides, macrolides, β-lactamases, efflux pumps, polymyxin resistance	Confirmed zoonotic agent	[14,52,53,54]
*B. mallei*	Equids, zoonotic to humans	Biofilm formation, motility, secretion systems, immune evasion, novel virulence proteins, modulation of ubiquitination, actin-cytoskeleton rearrangement	Aminoglycosides, β-lactams, efflux pumps	Confirmed zoonotic agent	[52,73,74,75]
*B. thailandensis*	Environment, immunocompromised hosts	Biofilm formation, motility, attenuated virulence, secretion systems, QS, siderophore (malleobactin) production	Limited resistance, efflux pumps, β-lactamases	Opportunistic zoonotic risk	[76,77,78]
*B. glumae*	Plants, rare human cases	Biofilm formation, motility, toxoflavin, lipase, QS, flagella, extracellular polysaccharides, lipase, secretion systems	Multidrug resistance, efflux pumps, β-lactamases	No known zoonotic transmission	[55,67,68,69]
*B. gladioli*	Plants, humans (CF, immunocompromised)	Biofilm formation, protein secretion systems (T2SS, T3SS), motility, proteases, toxoflavin, QS	β-lactams, aminoglycosides, multidrug efflux	Potential zoonotic pathogen	[70,71,72]

## 3. Characterization of *Burkholderia* Phages

### 3.1. Isolation

*Burkholderia* phages have been isolated from a wide range of environmental samples, including soil, water, plant tissues, compost, and clinical settings. Common isolation methods involve enrichment using selective media and plaque assays, where samples are mixed with host bacterial strains and plated onto agar to identify lytic and lysogenic phages through plaque formation [79,80]. For instance, Jungkhun et al. isolated 61 phages using direct plating and plaque assays, selecting NBP1-1, NBP4-7, and NBP4-8 as effective lytic agents against *B. glumae* [20]. Adachi et al. used filtration and ultracentrifugation to isolate phages BGPP-Ar, BGPP-Sa, and BGPP-Ya from water and puddles, demonstrating their potential for controlling bacterial seedling blight in rice [81]. Kanaizuka et al. obtained jumbo phages FLC8, FLC9, and FLC10 from fallen leaf compost, highlighting the natural abundance of *Burkholderia* phages in decaying plant material [82]. Jumbo phages *Chiangavirus* FLC6 and FLC8 infecting *B. glumae* were isolated from rice fields and compost samples [82,83]. *Lessievirus* BcepIL02 and *Aptresvirus* vB_BceM_AP3 infecting *B. cenocepacia* were obtained from soil sample planted with corns and irrigated fields [84,85]. These diverse isolations highlight the natural abundance and ecological adaptability of *Burkholderia* phages.

### 3.2. Morphology

Most *Burkholderia* phages possess icosahedral heads and exhibit either contractile or non-contractile (long or short) tails, morphologically classified into the families *Myoviridae*, *Podoviridae*, and *Siphoviridae*. For examples, jumbo *Burkholderia* phage FLC6 and non-jumbo *Burkholderia* phages NBP1-1, NBP4-7, and NBP4-8 infecting *B. glumae* possess icosahedral heads and contractile tails typical of the family *Myoviridae* [20,83]; *Burkholderia* phage Bp-AMP1 infecting *B. pseudomallei* has an icosahedral capsid and a short non-contractile tail typical of the family *Podoviridae*; *Burkholderia* phages phiE125 and phi1026b targeting *B. mallei* are characterized by icosahedral heads and long non-contractile tails typical of the family *Siphoviridae* [86,87,88]. Morphology-based phage classification depends on transmission electron microscopy to visualize phage particles and determine phage particle size, shape, and structural features [89].

### 3.3. Life Cycle

*Burkholderia* phages possess lytic or lysogenic life cycles (Table 2), impacting their use in phage therapy and biocontrol. Lytic *Burkholderia* phages hijack the host cellular machinery to replicate and lyse bacterial cells and are effective against pathogenic *Burkholderia*. For example, the jumbo phage *Chiangavirus FLC6* shows strong lytic activity against *B. glumae*, *B. plantarii*, and even *Ralstonia pseudosolanacearum*, indicating broad-host range and cross-genus infectivity [83]. While promising, this broad-host range requires further validation through in vivo studies and testing against diverse environmental isolates, as current evidence are mainly derived from in vitro assays. Lysogenic *Burkholderia* phages integrate their genome into the host genome as prophages [90,91]. This lysogenic conversion drives horizontal gene transfer and phage–host co-evolution, where integrated phage genes may enhance bacterial virulence, stress tolerance, or antibiotic resistance. Most characterized *Burkholderia* phages within the family *Peduoviridae*, such as *Kisquattuordecimvirus KS14*, *Kisquinquevirus KS5*, and *Tigrvirus phiE202*, are lysogenic (Table 2). Interestingly, *Ampunavirus* phage Bp-AMP1 has a temperature-dependent life cycle, remaining lysogenic at 25 °C but switching to a lytic cycle at 37 °C [92,93]. This thermally controlled behavior suggests its potential in temperature-regulated phage therapies. Although temperate phages have limited direct therapeutic use, synthetic biology allows for conversion into obligate lytic forms by disrupting lysogeny-related genes (e.g., integrases, repressors), expanding their clinical and agricultural applications [94]. Overall, lytic *Burkholderia* phages with broad-host ranges are promising for phage therapy. Temperate *Burkholderia* phages may require genetic modification or lytic derivative selection to ensure therapeutic safety and efficacy.

### 3.4. Host Range and Specificity

*Burkholderia* phages exhibit diverse host specificities, ranging from narrow-host to broad-host. Narrow-host phages infect very limited strains within one species, such as *Kayeltresvirus KL3* infecting only *B. ambifaria* LMG 17828, and temperate phages KS4 and KS9 infecting only two out of 24 tested Bcc strains. In contrast, broad-host phages can infect multiple bacterial species even genera, such as the jumbo phage FLC6, which can lyse multiple strains of *B. glumae*, *B. plantarii*, and *Ralstonia pseudosolanacearum* [83,90,95]. This specificity is primarily governed by tail fiber proteins (TFPs), which mediate phage–host interactions by recognizing bacterial surface receptors such as lipopolysaccharides (LPSs) and outer membrane proteins [96]. Variations in TFPs, including C-terminal extensions and single amino acid mutations, significantly impact host range [97]. Structural and genetic modifications in TFPs play a critical role in host adaptation. For example, *Burkholderia* phage AP3 possesses a unique 365-amino-acid C-terminal extension in its TFP that enhances its specificity for *B. cenocepacia* IIIA LPS variants, contributing to its narrow-host range [85]. Furthermore, engineered chimeric phages, such as *Pseudomonas aeruginosa* phage PaP1-rec1, acquire expanded host ranges through tail fiber gene swaps, demonstrating the potential of genetic modifications in customizing phage infectivity [98]. Recent advances, such as targeted point mutations (e.g., G→C in *Acinetobacter* phage Abp4-M) [99] and domain swapping (e.g., STyj5-1 with BD13 tail fibers) [100], have further expanded host ranges while maintaining adsorption efficiency. A rational therapeutic approach could involve phage cocktails, combining highly specific phages with engineered broad-range variants to balance efficacy and safety in treating multidrug-resistant infections.

### 3.5. Genomic Taxonomy

Bacteriophage taxonomy has evolved from a discipline based mainly on morphology to genome [101]. The morphology-based families *Myoviridae*, *Podoviridae*, and *Siphoviridae* were abolished and the order *Caudovirales* was replaced by the class *Caudoviricetes* to group all tailed bacterial and archaeal viruses with icosahedral capsids and a double-stranded DNA genome [102]. The advances of next-generation sequencing techniques promote the genome-based classification to generate a more accurate evolutionary framework and better reflection of the diversity and phylogeny of the abundant and diverse viruses, and establishment of new genome-based taxa recognized by the International Committee on Taxonomy of Viruses (ICTV) (Figure S1). Nowadays, ICTV uses a holistic approach to classify prokaryote viruses by considering morphotype, host, lifestyle, genome characteristics (such as size, mol% G + C), % protein homologs, overall DNA and protein similarity, and phylogeny based on core genes [101]. Prokaryote viruses belonging to the same taxonomy rank form a cohesive and monophyletic group. Two phages are assigned to the same species if their genomes are more than 95% identical at the nucleotide level over their full genome length, while 70% of nucleotide identity of the full genome length is the cut-off for genera [101]. Members of a viral family share a significant number of orthologous genes, forming a cohesive and monophyletic group based on common proteomes. The sequencing and analyzing of phage genomes revealed a much higher genomic diversity than had previously been considered, leaving a significant fraction of sequenced phages unclassified at the family level [101].

**Figure 1:**
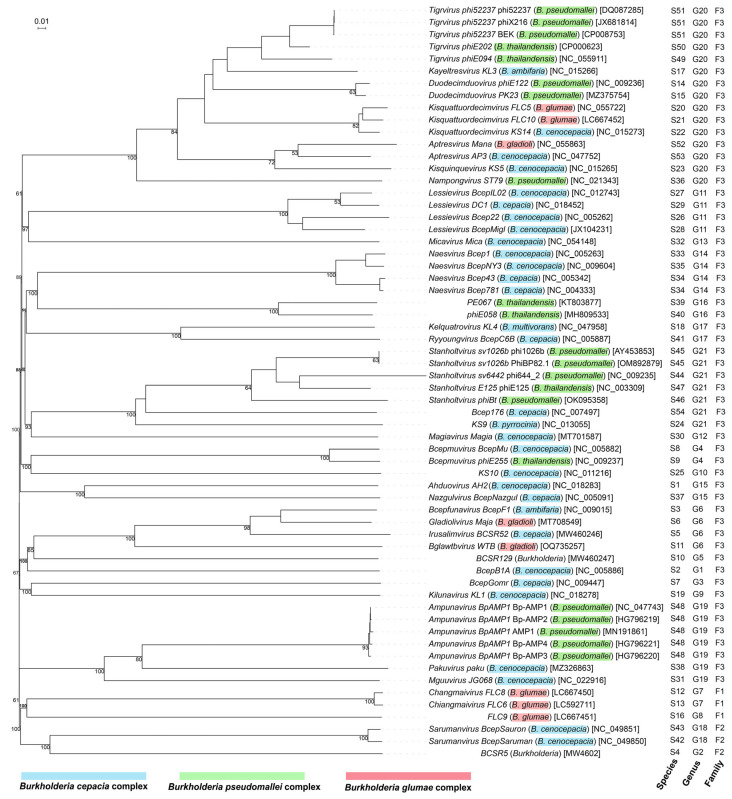
Phylogenomic relationships among *Burkholderia* phages within the class *Caudoviricetes*. The balanced minimum-evolution tree inferred from intergenomic distances based on whole-genome sequence comparisons was generated using the Genome-BLAST Distance Phylogeny (GBDP) method implemented in VICTOR [127]. Branch support was inferred from 100 pseudo-bootstrap replicates via FASTME including SPR postprocessing [128]. Taxon boundaries at the species, genus, and family level were estimated with the OPTSIL program [129], the recommended clustering thresholds [127], and an F value (fraction of links required for cluster fusion) of 0.5 [130]. The branch lengths are scaled in terms of the GBDP distance formula d0. The tree was rooted at the jumbo phages and displayed using the online tool iTOL version 7 (https://itol.embl.de/) (accessed on 16 June 2025). Tree leaves were labeled with phage names (host) [nucleotide sequence accession numbers in GenBank] and genomic classification of phages into species, genus, and family. Phage host species within *Burkholderia cepacia* complex, *Burkholderia pseudomallei* complex, and *Burkholderia glumae* complex are highlighted in blue, green, and red, respectively.

Almost all *Burkholderia* phages whose whole-genome sequences are deposited in the GenBank database of the National Center for Biotechnology Information (https://www.ncbi.nlm.nih.gov/) (accessed on 25 February 2025) belong to the class *Caudoviricetes*, except for *Alphatectivirus* BCE1, which belongs to the class *Tectiliviricetes* (Table 2). Another distinguished feature of *Alphatectivirus* BCE1 is the smallest genome size of 14,800 bp. Based on the genome size, *Burkholderia* phages belonging to the class *Caudoviricetes* are divided into two groups: jumbo *Burkholderia* phages and non-jumbo *Burkholderia* phages (Table 2, Figure 1). The genome size of the jumbo *Burkholderia* phages ranges from 225,545 bp to 321,833 bp. The genome size of the non-jumbo *Burkholderia* phages ranges from 32,090 bp to 72,415 bp.

**Table 2:** Information of *Burkholderia* phages.

Phage Name	Morphotype	ICTV Taxonomy (Class > Order > Family > Genus)	Host	Lifestyle	GC Content (%)	Genome Length (bp)	Reference
BCE1	/	*Tectiliviricetes > Kalamavirales > Tectiviridae* > *Alphatectivirus*	*B. cepacia*	/	48.21	14,800	[103]
		Class *Caudoviricetes*					
FLC6	Myovirus	*Chimalliviridae* > *Chiangmaivirus*	*B. glumae*; *B. plantarii*; *Ralstonia pseudosolanacearum*	Lytic	52.01	227,105	[83]
FLC8	Myovirus	*Chimalliviridae* > *Chiangmaivirus*	*B. glumae*; *B. plantarii*	Lytic	52.05	225,545	[82]
S13	Myovirus	*Chimalliviridae* > *Chiangmaivirus*	*B. glumae*; *B. gladioli*; *B. multivorans*; *B. cenocepacia*; *B. dolosa*;	Lytic	51.7	227,647	[21]
FLC9	Myovirus	Novel species 16 within a novel genus 8 *	*B. glumae*; *B. plantarii*	/	55.97	321,833	[82]
BcepSauron	Myovirus	*Sarumanvirus*	*B. cenocepacia*	Lytic	58.10	262,653	[104]
BcepSaruman	Myovirus	*Sarumanvirus*	*B. cenocepacia*	Unknown	58.14	263,735	/
BCSR5	Myovirus	Novel species 4 within a novel genus 2 *	*B. cepacia*	*/*	54.74	227,351	[105]
KL1	Siphovirus	*Jondennisvirinae* > *Kilunavirus*	*B. cenocepacia*	Lytic	54.61	42,832	[106]
BcepGomr	/	Novel species 7 within a novel genus 3 *	*Burkholderia*	Unknown	56.29	52,414	[106]
Bp-AMP2	Podovirus	*Autographivirales* > *Autonotataviridae* > *Ampunavirus*	*B. pseudomallei*	/	61.76	42,492	[92]
Bp-AMP1	Podovirus	*Autographivirales > Autonotataviridae > Ampunavirus*	*B. pseudomallei*; *B. thaliandensis*	Temperate	61.75	42,409	[92,93]
Bp AMP4	Podovirus	*Autographivirales > Autonotataviridae > Ampunavirus*	*B. pseudomallei*	/	61.79	42,112	[92]
Bp AMP3	Podovirus	*Autographivirales > Autonotataviridae > Ampunavirus*	*B. pseudomallei*	/	61.77	41,882	[92]
JG068	Podovirus	*Autographivirales > Autonotataviridae > Mguuvirus*	*B. multivorans*; *B. cenocepacia*; *B. stabilis*; *B. dolosa*	Lytic	60.69	41,604	[107]
Paku	/	*Autographivirales* > *Autonotataviridae* > *Pakuvirus*	*B. cenocepacia*	Temperate	61.86	42,727	[107]
Maja	Myovirus	*Lindbergviridae > Gladiolivirus*	*B. gladioli*	Temperate	54.50	68,393	[108]
BcepF1	Myovirus	*Lindbergviridae > Bcepfunavirus*	*B. ambifaria*	*/*	55.89	72,415	[106,109]
BCSR52	Myovirus	*Lindbergviridae > Irusalimvirus*	*B. cepacia*	*/*	51.45	70,038	/
WTB	Myovirus	*Bglawtbvirus*	*B. gladiol* *i*	Lytic	60.04	68,541	[110]
BCSR129	Myovirus	Novel species 10 within a novel genus 5 *	*B. cepacia*	Unknown	58.42	66,147	[105]
BcepB1A	Myovirus	Novel species 2 within a novel genus 1 *	*B. cenocepacia*	Lytic	54.45	47,399	[106]
BcepNazgul	Siphovirus	*Casjensviridae* > *Nazgulvirus*	*B. cepacia*	Lytic	60.64	57,455	[111]
AH2	Siphovirus	*Casjensviridae* > *Ahduovirus*	*B. cenocepacia*; *B. gladioli*	Lytic	61.31	58,065	[106,112]
PhiE255	Myovirus	*Bcepmuvirus*	*B. thailandensis*	Temperate	63.05	37,446	[91]
BcepMu	Myovirus	*Bcepmuvirus*	*B. cenocepacia*	Temperate	62.86	36,748	[18]
KS10	Myovirus	Novel species 25 within a novel genus 10 *	*B. cenocepacia*; *B. stabilis*; *B. ambifaria*	Temperate	62.87	37,635	[113]
phiX216	Myovirus	*Peduoviridae* > *Tigrvirus*	*B. pseudomallei*; *B. mallei*	Temperate	64.82	37,637	[114]
phi52237	Myovirus	*Peduoviridae* > *Tigrvirus*	*B. pseudomallei*	Temperate	64.82	37,639	[91]
BEK	Myovirus	*Peduoviridae* > *Tigrvirus*	*B.* *pseudomallei*	/	68.82	37,631	[85]
phiE202	Myovirus	*Peduoviridae* > *Tigrvirus*	*B. mallei*; *B. pseudomallei*	Temperate	65.43	35,741	[91]
phiE094	Myovirus	*Peduoviridae* > *Tigrvirus*	*B. thailandensis*; *B. pseudomallei*	Temperate	64.48	37,727	[115]
NBP1-1	Myovirus	*Peduoviridae* > *Tigrvirus*	*B. glumae*	Lytic	63.23	40,570	[20]
NBP4-7	Myovirus	*Peduoviridae* > *Tigrvirus*	*B. glumae*	Lytic	63.23	40,563	[20]
NBP4-8	Myovirus	*Peduoviridae* > *Tigrvirus*	*B. glumae*	Lytic	63.23	40,568	[20]
KL3	Myovirus	*Peduoviridae* > *Kayeltresvirus*	*B. ambifaria*	Temperate	63.23	40,555	[90]
PK23	Myovirus	*Peduoviridae* > *Duodecimduovirus*	*B. pseudomallei*	Temperate	65.12	35,343	[116]
phiE12_2	Myovirus	*Peduoviridae* > *Duodecimduovirus*	*B. mallei*	Temperate	64.62	36,690	[91]
FLC10	Myovirus	*Peduoviridae > Kisquattuordecimvirus*	*B. glumae*	Lytic	61.29	32,867	[82]
FLC5	Myovirus	*Peduoviridae > Kisquattuordecimvirus*	*B. glumae*; *B. plantarii*	Temperate	61.79	32,090	[117]
KS14	Myovirus	*Peduoviridae > Kisquattuordecimvirus*	*B. multivorans*; *B. cenocepacia*; *B. dolosa*; *B. ambifaria*	Temperate	62.28	32,317	[90]
vB BceM AP3	Myovirus	*Peduoviridae > Aptresvirus*	*B. cenocepacia*	Temperate	64.04	36,499	[85]
Mana	Myovirus	*Peduoviridae > Aptresvirus*	*B. gladioli*	/	64.31	38,038	[118]
KS5	Myovirus	*Peduoviridae > Kisquinquevirus*	*B. multivorans*; *B. cenocepacia*	Temperate	63.71	37,236	[90]
ST79	Myovirus	*Peduoviridae > Nampongvirus*	*B. pseudomallei*; *B. mallei*	Lytic	62.50	35,430	[119]
BcepMigl	Podovirus	*Lessievirus*	*B. cenocepacia*	/	65.51	62,952	/
Bcep22	Podovirus	*Lessievirus*	*B. cenocepacia*	Temperate	65.31	63,882	[84]
DC1	Podovirus	*Lessievirus*	*B. cepacia*; *B. cenocepacia*; *B. stabilis*	Temperate, unstably lysogenic	66.21	61,847	[120]
BcepIL02	Podovirus	*Lessievirus*	*B. cenocepacia*	Temperate	66.20	62,715	[84]
Mica	Myovirus	*Micavirus*	*B. cenocepacia*	Temperate	62.15	43,707	[121]
Bcep781	Myovirus	*Naesvirus*	*B. cepacia*	Lytic	63.33	48,247	[122]
Bcep43	Myovirus	*Naesvirus*	*B. cepacia*	Lytic	63.43	48,024	[122]
BcepNY3	/	*Naesvirus*	*B. cenocepacia*	/	63.64	47,382	/
Bcep1	Myovirus	*Naesvirus*	*B. cenocepacia*	Lytic	63.64	48,177	[122]
phiE058	Myovirus	Novel species 40 within a novel genus 16 *	*B. mallei*; *B. pseudomallei*; *B. thailandensis*	Temperate	64.12	44,121	[123]
PE067	Myovirus	Novel species 39 within a novel genus 16 *	*B. pseudomallei*; *B. thailandensis*	Temperate	64.48	43,649	[123]
BcepC6B	Podovirus	*Ryyoungvirus*	*B. cepacia*	Temperate	65.19	42,415	[122]
vB BmuP KL4	/	*Kelquatrovirus*	*B. multivorans*	/	63.18	42,250	/
Magia	Myovirus	*Magiavirus*	*B. cenocepacia*	Temperate	65.06	44,942	[124]
phiE125	Siphovirus	*Stanholtvirus*	*B. mallei*	Temperate	61.19	53,373	[86]
Phi644_2	Siphovirus	*Stanholtvirus*	*B. mallei*; *B. pseudomallei*	Temperate	60.45	48,674	[91]
PhiBP82.1	/	*Stanholtvirus*	*B. pseudomallei*	/	60.68	54,921	/
Phi1026b	Siphovirus	*Stanholtvirus*	*B. mallei*; *B. pseudomallei*	Temperate	60.68	54,865	[87]
phiBt	/	*Stanholtvirus*	*B. pseudomallei*	/	60.30	56,453	/
Bcep176	Siphovirus	*Stanholtvirus*	*B. multivorans*; *B. cepacia*	Temperate	61.54	44,856	[125]
KS9	Siphovirus	*Stanholtvirus*	*B. pyrrocinia*; *B. cenocepacia*	Temperate	60.68	39,896	[18,126]

* Genomic classification by VICTOR [127] in Figure 1; / data unavailable.

Sixty-one whole-genome sequences of *Burkholderia* phages within the class *Caudoviricetes* were used to generate a phylogenomic tree using the Genome BLAST Distance Phylogeny (GBDP) method implemented in VICTOR (https://ggdc.dsmz.de/victor.php) (accessed on 26 February 2025) [127], allowing genome-based classification. The 61 *Burkholderia* phages are classified into 54 species, 21 genera, and 3 families (Figure 1). *Family 1* includes the jumbo *Burkholderia* phages *Chiangmaivirus* FLC6 and FLC8 within the ICTV family *Chimalliviridae* and an unclassified genus (phage FLC9), which infect *B. glumae*. *Family 2* includes the jumbo *Burkholderia* phage *Sarumanvirus* infecting *B. cenocepacia* and an unclassified genus (phage BCSR5). *Family 3* includes all non-jumbo *Burkholderia* phages belonging to 17 genomogenera, among which 8 genera were classified into 5 existing ICTV families (*Jondennisvirinae*, *Autonotataviridae*, *Lindbergviridae*, *Casjensviridae*, and *Peduoviridae*). In other words, one genome-based family contains all non-jumbo *Burkholderia* phages within the class *Caudoviricetes*, indicating a limited taxon range of the non-jumbo *Burkholderia* phages. Nonetheless, the G + C mol% of these non-jumbo *Burkholderia* phages ranging from 51.45% to 68.82% indicates considerable genomic diversities within the genome-based *Family 3*. Together, this phylogenomic overview highlights both evolutionary divergence and taxonomic coherence among *Burkholderia* phages.

The VICTOR phylogenomic overview (Figure 1) also shows the host ranges at three phage taxon levels. First, the host range of the *Burkholderia* phages within *Family 3* covers the genus *Burkholderia sensu stricto*. Second, multiple *Burkholderia* phages within a virus species infects only one *Burkholderia* species. For example, four *Burkholderia* phages within *Ampunavirus BpAMP1* infect *B. pseudomallei*; two *Burkholderia* phages within *Stanholtvirus sv1026b* infect *B. pseudomallei*; three *Burkholderia* phages within *Tigrvirus phi52237* infect *B. pseudomallei*; and *Naesvirus Bcep781* and *Naesvirus Bcep43* composing a genomospecies infect *B. cepacia*. Third, multiple virus species within multiple genera can infect the same *Burkholderia* species. As just noted, *Ampunavirus BpAMP1*, *Stanholtvirus sv1026b*, and *Tigrvirus phi52237* infect *B. pseudomallei*. Fourth, multiple virus genera can infect the same multiple species within a species complex. For example, *Lessievirus* and *Naesvirus* infect Bcc species *B. cepacia* and *B. cenocepacia*; *Nazgulvirus BcepNazgul* and *Ahduovirus AH2* composing a genomogenus also infect *B. cepacia* and *B. cenocepacia*. Fifth, a virus genus can infect multiple *Burkholderia* species within multiple species complexes. For example, *Bcepmuvirus* infects *B. thailandensis* (Bpc) and *B. cenocepacia* (Bcc); *Ampunavirus BpAMP1*, *Pakuvirus paku*, and *Mguuvirus JG068* composing a genomogenus infect *B. pseudomallei* (Bpc) and *B. cenocepacia* (Bcc); *Gladiolivirus Maja*, *Bcepfunavirus BcepF1*, *Irusalimvirus BCSR52*, and *Bglawtbvirus WTB* composing a genomogenus infect *B. cepacia* (Bcc), *B. ambifaria* (Bcc), and *B. gladioli* (Bgc). *Tigrvirus*, *Kayeltresvirus*, *Duodecimduovirus*, *Kisquattuordecimvirus*, *Aptresvirus*, *Kisquinquevirus*, and *Nampongviru*s composing a genomogenus infect *B. pseudomallei* (Bpc), *B. thailandensis* (Bpc), *B. cenocepacia* (Bcc), *B. ambifaria* (Bcc), *B. glumae* (Bgc), and *B. gladioli* (Bgc).

Together, the genetic variability of the *Burkholderia* phages holds significant promise for both medical and agricultural applications. The multiple virus species or genera targeting the same *Burkholderia* species or species complex supports the strategy of using phage cocktails to control the *Burkholderia*-associated human, animal, or plant diseases. The phage cocktails containing diverse *Burkholderia* phages may use multiple mechanisms to control *Burkholderia* pathogens and to avoid immune escape by the *Burkholderia* pathogens.

## 4. Mechanism of Phage Action and *Burkholderia* Resistance

Phages infect *Burkholderia* host cells by recognizing and adsorbing to surface receptors, primarily LPS on the outer membrane. LPS consists of Lipid A (anchored in the membrane and responsible for endotoxicity), a core oligosaccharide with conserved inner and variable outer regions, and a highly variable O-antigen polysaccharide chain [21]. Additional receptors include capsular polysaccharides, flagella, and fimbriae [116]. After injection of genomes, phages hijack bacterial machinery to replicate and produce holins (membrane pore-forming proteins) and endolysins (peptidoglycan-degrading enzymes), which disrupt the cell envelope, leading to lysis and release of progeny phages [15,131].

**Figure 2:**
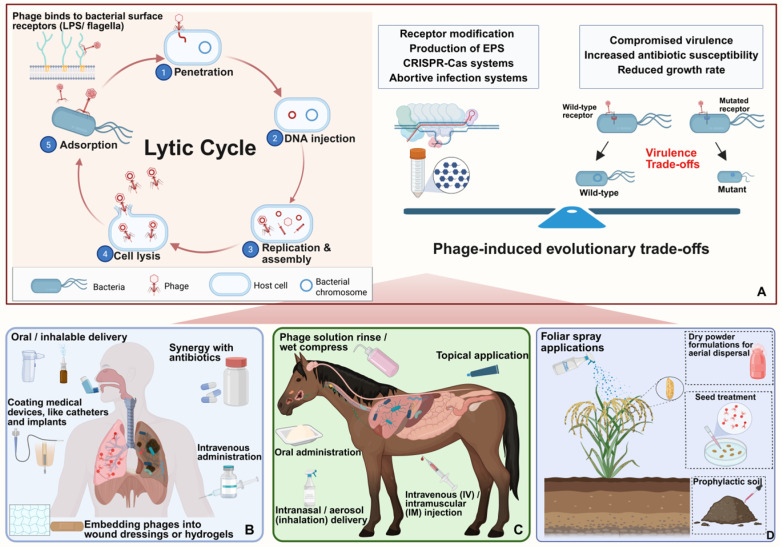
Mechanisms and application of *Burkholderia* phages in control of human, animal, and plant diseases. (**A**) Mechanism and evolutionary trade-offs: The lytic cycle—from adsorption to lysis—eliminates bacteria cells. In response, bacteria evolve resistance mechanisms such as receptor modification and CRISPR-Cas systems, which incur fitness costs and can increase sensitivity to antibiotics. (**B**) Human therapy: Phages are administered via oral, inhalable, intravenous, and topical routes. Phages can be used with antibiotics and medical device coatings. (**C**) Veterinary use: Phages are delivered via oral, inhalable, intravenous, intramuscular and topical routes. (**D**) Agricultural application: Phages are applied via foliar sprays, soil treatments, or seed coatings to control plant diseases.

Phage predation drives bacterial resistance primarily through modifications or loss of receptors. However, bacterial surface components are also critical to bacterial survival, motility, or virulence. As a result, receptor modifications frequently incur fitness costs including increased susceptibility to host immune factors and antibiotics [132]. For example, *B. cenocepacia* mutants with truncated LPS exhibit phage resistance but compromise serum resistance and increase sensitivity to colistin [133]. Similarly, infection by phage Bp-AMP1 can downregulate efflux pumps in *B. thailandensis*, increasing bacterial sensitivity to a broad range of antibiotics [134]. Beyond receptor modifications, *Burkholderia* has additional phage defense mechanisms, including excessive production of extracellular polysaccharides to physically shield receptors, activation of CRISPR-Cas systems to destroy invading phage genomes, and abortive infection systems that trigger programmed cell death to prevent phage propagation. However, these strategies also incur fitness trade-offs: overproduction of extracellular polysaccharide, reducing motility and nutrient uptake; CRISPR-Cas systems requiring metabolic resources and carrying a risk of autoimmunity; and abortive infection sacrificing the survival of individual cells [135,136]. These fitness trade-offs form the foundation of “phage steering” [133]. Strategically, phage–antibiotic synergy and phage therapy using phage cocktails can reduce bacterial resistance development and improve therapeutic outcomes. Combining phages with antibiotics such as meropenem can enhance bacterial clearance, reduce antibiotic doses, and delay resistance development [137]. Phage cocktails targeting diverse bacterial receptors and using evolving phages through directed adaptation improves treatment efficacy and mitigates *Burkholderia* resistance development (Figure 2).

## 5. Biotechnological Applications of *Burkholderia* Phages

The success of phage therapy against *Burkholderia* infections relies heavily on the selection of appropriate delivery strategies that can overcome biological and environmental barriers across different systems: humans, animals, and plants.

### 5.1. Medical and Veterinary Applications

In human medicine, phages targeting *Burkholderia* species are primarily administered via inhalation or intravenous injection, tailored to infection sites. Aerosolized delivery, especially through nose-only inhalation devices, has demonstrated efficacy in murine models by significantly reducing lung bacterial loads caused by *B. cenocepacia* [18]. This method provides direct access to the respiratory tract, a common infection site in CF patients, and ensures phage viability post-aerosolization despite mechanical and pH stress [133]. In clinical cases, intravenous phage therapy, such as the administration of phage BdPF16phi4281, has been used compassionately to treat *B. dolosa* infections, resulting in temporary bacterial load reductions [138]. However, systemic administration poses risks of immune clearance and antibiotic-related toxicity, underscoring the need for improved delivery formulations.

In veterinary medicine, although *Burkholderia*-specific phages are yet to be tested, analogs targeting other pathogens like *Salmonella* have shown promising outcomes via oral and topical delivery in broilers [139]. These methods provide scalable, practical models for future adaptation to treat *Burkholderia* infections in livestock, especially for gastrointestinal or dermal infections.

### 5.2. Agricultural Applications

Phage-based biocontrol presents a sustainable alternative to chemical pesticides for managing *Burkholderia*-associated plant diseases. Effective deployment, however, requires consideration of environmental factors such as UV exposure, high temperature, desiccation, and phage persistence in the phyllosphere and rhizosphere [23]. Several *Burkholderia*-specific phages have shown potential in agricultural disease management. Phages KS12 and AH2, targeting *B. gladioli*, significantly reduce tissue destruction in onion and mushroom using a quantitative ex planta maceration model [112]. Phage WTB (vB_BglM_WTB), a high-efficiency lytic phage, also targets *B. gladioli*, offering rapid suppression of infections and potential for field deployment [110]. For *B. glumae*, a key pathogen of rice, the jumbo phage S13 demonstrates a unique flagella-dependent infection mechanism. By selecting for non-flagellated, less virulent mutants, S13 reduces pathogenicity while directly lysing motile bacterial populations [21]. Similarly, compost-derived jumbo phages FLC8 and FLC9 display broad-host ranges and have achieved over 77% control of rice seedling rot in greenhouse assays, while FLC10 exhibits narrower efficacy [82]. Application methods for these phages vary based on the plant–pathogen context. Foliar sprays, commonly used against epiphytic pathogens, are suitable for applying phages like KS12 and AH2 to aerial plant parts. However, foliar applications of phages are vulnerable to rapid UV inactivation; phage viability may drop below 1% within hours under sunlight [140]. To address this problem, formulations with UV-protective agents and humectants are being developed to enhance phage persistence on leaves. Soil drenching offers an effective alternative for root-associated infections by delivering phages like FLC8, FLC9, and FLC10 directly to the rhizosphere. This approach exploits phage mobility in moist soil, improving contact with root pathogens [141]. Additionally, seed coating with phages, particularly using polymer-based carriers, provides early-stage protection during germination and colonization of the rhizosphere, enhancing defense against soil-borne *Burkholderia* [142].

### 5.3. Nanotechnology-Enhanced Delivery

Nanotechnology-based delivery systems are increasingly used in phage therapy to enhance survival, targeting, and controlled release of phages [139,143,144,145,146,147,148,149,150,151,152,153,154,155,156,157]. Alginate and chitosan nanocarriers, leveraging their pH-responsive properties, effectively protect phages during gastrointestinal transit while promoting mucosal adhesion. This makes them ideal for oral delivery in humans and animals, as they shield phages from gastric acidity and enable targeted intestinal release [143]. However, most studies remain at the in vitro or proof-of-concept stage, and more in vivo validation is required.

Hydrogel matrices, such as alginate–CaCO_3_ microcapsules, provide sustained phage release and have demonstrated efficacy in veterinary models by maintaining anti-*Salmonella* activity in poultry [139]. Their adaptability suggests potential application for *Burkholderia*-specific phages targeting both respiratory and gastrointestinal infections, though direct evidence for these specific phages is limited to date.

Other nanocarrier systems, such as liposomes, polymeric nanoparticles, nanofibers, and whey protein isolate-based films, expand phage therapy’s utility in clinical and agricultural settings [144,145,146,147]. These systems improve phage stability, controlled release, and adhesion to biological or environmental surfaces. Liposome encapsulation, for instance, protects phages in respiratory infections but requires optimization to address immune clearance and limited systemic circulation [148]. Notably, whey protein isolate-based films, especially when reinforced with chitosan nanofibers or nano-chitin, form biodegradable and biocompatible matrices ideal for encapsulating phages [149,150]. These composite systems support long-term storage, pH-responsive release, and enhanced adhesion, making them promising candidates for bioactive seed coatings and durable phage packaging in agricultural applications (Table 3).

## 6. Conclusions and Perspectives

The convergence of *Burkholderia* pathogens infecting plants, animals, and humans highlights their significance within the One Health framework. Some *Burkholderia* species exhibit cross-kingdom infectivity and share resistance mechanisms, such as efflux pumps, quorum sensing-regulated virulence, and biofilm formation. The zoonotic potential of Bpc species and the increasing clinical detection of Bcc strains from environmental and animal reservoirs emphasize the interconnectedness of ecosystems [5,45].

Lytic *Burkholderia* phages offer the foundation for phage therapy targeting *Burkholderia*-associated diseases in humans, animals, and plants. Genetically distinct *Burkholderia* phages belonging to different genera or even different families target the same *Burkholderia* species or multiple *Burkholderia* species causing the same disease, providing nature resources for phage cocktails with reduced risk of immune escape and resistance emergence. While temperate phages may be used after modification, either through the selection of lytic derivatives or synthetic design, advances in synthetic biology allow for the engineering of phages with defined host ranges, enabling a balance between therapeutic efficacy and biosafety against multidrug-resistant infections. Moreover, phage–antibiotic synergy can also stimulate increased phage activity and reduce the risk of resistance emergence, making it a valuable complement to phage cocktail strategy and synthetic biology in combating multidrug-resistant *Burkholderia* infections.

Effective phage therapy requires targeted delivery, environmental stability, and sustained activity. Appropriate delivery strategies can overcome biological and environmental barriers specific to each *Burkholderia*–host system. Lessons from clinical and veterinary applications, such as mucoadhesive polymers for gastrointestinal use and liposome encapsulation for respiratory infections, can be adapted for agricultural purposes. Encapsulation methods like alginate microbeads and alginate/chitosan composites protect phages from environmental stress and allow for controlled pH-responsive release. These formulations are compatible with diverse agricultural delivery modes, including foliar sprays, soil drenches, and seed coatings. Moreover, spray-dried phage powders and electrospun nanofiber matrices enable the production of stable, field-ready products. The natural mucoadhesive and biodegradable properties of alginate and chitosan enhance phage targeting and prolong antibacterial activity. Whey protein isolate-based films, especially those reinforced with chitosan nanofibers or nano-chitins, offer biodegradable and biocompatible matrices for long-term storage and sustained bioactivity.

Together, these nanotechnology-enabled delivery systems bridge the gap between laboratory research and real-world implementation of phage therapy. The development of controlled host range phage cocktails, refined application-specific delivery systems, field trials, and regulatory frameworks holds promises for establishing robust, sustainable, and scalable phage-based biocontrol strategies across various *Burkholderia*–host systems. This integrated approach aligns closely with the One Health perspective, offering an eco-friendly alternative to antibiotics and chemical pesticides for managing *Burkholderia*-associated diseases in clinical and agricultural contexts.

## Figures and Tables

**Table 3:** Nanotechnology-enhanced phage delivery strategies.

Nanocarrier Type	Mechanism/Function	Human/Veterinary Use	Potentials in Agriculture	Translational Insight	References
Alginate/Chitosan	pH-responsive protection; mucosal adhesion	Oral delivery in gastrointestinal infections	Seed coating or root-targeted release	Protect phages during transit through acidic environments: adaptable to rhizosphere targeting	[143]
Hydrogels like Alginate–CaCO_3_	Sustained, slow release over time	Poultry models for *Salmonella* control	Soil drenching or foliar application	Long-lasting effect under variable field conditions; ideal for crop protection	[139]
Liposomes	Encapsulation for enhanced penetration	Oral delivery for gastrointestinal infections	Not yet applied	Protect phages from acid and enzymes while enabling slow release	[145]
Polymeric nanoparticles	Precision targeting; immune evasion	Under development	Experimental in agriculture	Enhance nanoparticle uptake by plant cells through foliar spray or irrigation water delivery to plant tissues	[146]
Nanofibers	High surface area; controlled release and adhesion	Wound dressing, tissue scaffolds for drug delivery	Leaf surface coating or seed coating	Provide gradual phage release, enhance adhesion to plant surfaces, and improve stability	[147]
Whey protein isolate-based films	Biopolymer matrix for encapsulation; moisture barrier, and controlled release	Not yet applied clinically; explored for probiotic and drug delivery	Edible coating, seed wraps, and phage packaging for crops	Enhance phage storage stability and enable slow release. Integration with nanofibers, chitosan, or nano-chitin expands potential for agricultural delivery systems	[150,151]
DL-lactic-co-glycolic acid microspheres (PLGA)	Encapsulate lyophilized (freeze-dried) phages for controlled release and protection	Biocompatible and approved for human use like inhalable phage delivery	Foliar or root delivery; greenhouse applications	Biodegradable, biocompatible, and tunable degradation rates (via lactide/glycolide ratios) for sustained phage release in crops	[152,153,154]
Lactose/lactoferrin 60:40 (*w*/*w*)	Carrier matrix for dry powder phage formulations; enhance stability and dispersibility	Used in inhalable dry powder formulations for pulmonary phage therapy	Spray-dried phage powders for crop protection	Protect phages during drying and storage; potential for integration into foliar sprays	[155]

## Data Availability

The original contributions presented in this study are included in the article/Supplementary Material. Further inquiries can be directed to the corresponding authors.

## Supplementary Materials

The following supporting information can be downloaded at: https://www.mdpi.com/article/10.3390/microorganisms13081873/s1, Table S1. Pathogenic *Burkholderia* species. Figure S1. Comparison of morphology-based and genome-based phage classification. Conventional morphology-based phage taxonomy classifies phages by capsid shape and tail type using electron microscopy. Modern genome-based phage taxonomy classifies phages by genome sequencing, overall DNA and protein similarity, and phylogenetic analyses based on core genes and proteins. This transition improves classification in capturing phage diversity and evolutionary relationships. References [158,159,160,161,162,163,164,165,166,167,168,169,170,171,172,173,174,175,176,177] are cited in the Supplementary Materials.
