# Supplementary material for: Burkholderia Phages and Control of Burkholderia-Associated Human, Animal, and Plant Diseases
Source: Microorganisms. 2025 Aug 11;13(8):1873. doi: 10.3390/microorganisms13081873 (PMC12388082; doi:10.3390/microorganisms13081873)
Supplement: Supplementary file 1 [file microorganisms-13-01873-s001.zip › microorganisms-3745412-supplementary.pdf]

Table S1. Pathogenic *Burkholderia* species

Burkholderia	**Host**	**Disease**	**Reference**
Burkholderia cepacia complex			
B. cepacia	Human / Animals / Plants	Respiratory infections in cystic fibrosis (CF) / Sepsis; dermatitis / Bulb rot of onions; banana finger-tip rot	[1-4]
B. multivorans	Human	Lung infections in CF patients; sepsis and pneumonia in chronic granulomatous disease patients; bacterial meningitis	[5, 6]
B. cenocepacia	Human	Lung infections, necrotizing pneumonia and septicemia in CF patient	[7]
B. stabilis	Human	Bloodstream infections, noninvasive infections, and wound contaminations	[8, 9]
B. vietnamiensis	Human	Bacteremia, pulmonary infections	[10, 11]
B. dolosa	Human	Necrotizing pneumonia, accelerated decline in pulmonary function and increased mortality	[12, 13]
B. ambifaria	Human	Lung infections in immunocompromised patients and those with CF	[14]
B. anthina	Human	Chronic obstructive pulmonary diseases in CF patients	[15, 16]
B. pyrrocinia	Human	Lung infections in CF patients	[17, 18]
B. contaminans	Human	Respiratory tract colonization and chronic infection in CF patients	[19-22]
B. lata	Human	Healthcare-associated infection outbreaks	[23]
B. pseudomultivorans	Animals	Companion animals (cat) with sepsis	[24, 25]
B. orbicola	Plants	Bean seed germination reduction and impact *Galleria mellonella* wax moth larvae survival	[26]
B. semiarida	Plants	Onion sour skin	[27]
B. sola	Plants	Onion sour skin	[27]
Burkholderia pseudomallei complex			
B. pseudomallei	Human / Animals	Melioidosis – zoonotic disease, affects animals including goats, sheep, and pigs	[28-30]
B. mallei	Human / Animals	Glanders – zoonotic disease, affects equids like horses, mules, and donkeys	[31-33]
B. thailandensis	Human	Melioidosis; pneumonia; septicemia	[34-36]
B. oklahomensis	Human	Wound ulcer	[37]
B. singularis	Human	Opportunistic pathogen for CF patients	[38]
Burkholderia glumae complex			
B. glumae	Plants / Human	Bacterial panicle blight in rice; bulb rot in onion / Infection in an infant with chronic granulomatous disease	[39-41]
B. gladioli	Plants / Human	Bacterial panicle blight in rice; scape blight in gladiolus bulb rot in onion, iris, and gladiolus; leaf spot or soft rot on vegetables and ornamental plants like lettuce, leek, tulips / Infection in CF patients following lung transplantation	[42-46]
B. plantarii	Plants	Grain and seedling rot	[47]

References

1. Burkholder, W. H. Sour skin, a bacterial rot of onion bulbs. *Phytopathology* **1950**, 40, 115-117.
2. Thomassen, M.; Demko, C.; Klinger, J.; Stern, R., *Pseudomonas cepacia* colonization among patients with cystic fibrosis: a new opportunist. *Am. Rev. Respir. Dis.* **1985,** 131, 791-796.
3. Zhang, Y.; Liu, F.; Wang, B.; Qiu, D.; Liu, J.; Wu, H.; Cheng, C.; Bei, X.; Lü, P., First report of *Burkholderia cepacia* causing finger-tip rot on banana fruit in the Guangxi province of China. *Plant Dis.* **2022,** 106, 1979.
4. Cain, C. L.; Cole, S. D.; Bradley II, C. W.; Canfield, M. S.; Mauldin, E. A., Clinical and histopathological features of *Burkholderia cepacia* complex dermatitis in dogs: a series of four cases. *Vet. Dermatol.* **2018,** 29, 457-e156.
5. Epithelia, W.-D. H. A., Role of Actin Filament Network in *Burkholderia multivorans* Invasion in Well-Differentiated Human Airway Epithelia. *Infect. Immun.* **2003**, 6607-6609.
6. Peralta, D. P.; Chang, A. Y.; Ariza-Hutchinson, A.; Ho, C. A., Burkholderia multivorans: A rare yet emerging cause of bacterial meningitis. *IDCases* **2018,** 11, 61-63.
7. Mahenthiralingam, E.; Urban, T. A.; Goldberg, J. B., The multifarious, multireplicon *Burkholderia cepacia* complex. *Nat. Rev. Microbiol.* **2005,** 3, 144-156.
8. Seth-Smith, H. M.; Casanova, C.; Sommerstein, R.; Meinel, D. M.; Abdelbary, M. M.; Blanc, D. S.; Droz, S.; Führer, U.; Lienhard, R.; Lang, C., Phenotypic and genomic analyses of *Burkholderia stabilis* clinical contamination, Switzerland. *Emerging Infect. Dis.* **2019,** 25, 1084.
9. Hudson, M. J., Outbreak of *Burkholderia stabilis* infections associated with contaminated nonsterile, multiuse ultrasound gel—10 states, May–September 2021. *MMWR Morb. Mortal. Wkly Rep.* **2022,** 71, 1517–1521.
10. Kar, M.; Dubey, A.; Sahu, C.; Patel, S. S., *Burkholderia vietnamiensis* causing bacteremia in patients suffering from B-Cell acute lymphocytic leukemia: a case series and review of literature. *J. Lab. Physicians* **2023,** 16, 134-139.
11. Flores-Vega, V. R.; Lara-Zavala, B. A.; Jarillo-Quijada, M. D.; Fernández-Vázquez, J. L.; Alcántar-Curiel, M. D.; Vargas-Roldán, S. Y.; Ares, M. A.; de la Cruz, M. A.; Morfín-Otero, R.; Rodríguez-Noriega, E., *Burkholderia vietnamiensis* causing infections in noncystic fibrosis patients in a tertiary care hospital in Mexico. *Diagn. Microbiol. Infect. Dis.* **2023,** 105, 115866.
12. Roux, D.; Weatherholt, M.; Clark, B.; Gadjeva, M.; Renaud, D.; Scott, D.; Skurnik, D.; Priebe, G. P.; Pier, G.; Gerard, C., Immune recognition of the epidemic cystic fibrosis pathogen *Burkholderia Dolosa*. *Infect. Immun.* **2017,** 85, e00765-16.
13. Kalish, L. A.; Waltz, D. A.; Dovey, M.; Potter-Bynoe, G.; McAdam, A. J.; LiPuma, J. J.; Gerard, C.; Goldmann, D., Impact of *Burkholderia dolosa* on lung function and survival in cystic fibrosis. *Am. J. Respir. Crit. Care Med.* **2006,** 173, 421-425.
14. Vial, L.; Groleau, M.-C.; Lamarche, M. G.; Filion, G.; Castonguay-Vanier, J.; Dekimpe, V.; Daigle, F.; Charette, S. J.; Déziel, E., Phase variation has a role in *Burkholderia ambifaria* niche adaptation. *ISME J.* **2010,** 4, 49-60.
15. Alshiekheid, M. A.; Dou, A. M.; Algahtani, M.; Al-Megrin, W. A. I.; Alhawday, Y. A.; Alradhi, A. E.; Bukhari, K.; Alharbi, B. F.; Algefary, A. N.; Alhunayhani, B. A., Bioinformatics and immunoinformatics assisted multiepitope vaccine construct against *Burkholderia anthina*. *Saudi Pharm. J.* **2024,** 32, 101917.
16. Pham, A.; Volmer, J. G.; Chambers, D. C.; Smith, D. J.; Reid, D. W.; Burr, L.; Wells, T. J., Genomic analyses of *Burkholderia* respiratory isolates indicates two evolutionarily distinct *B. anthina* clades. *Front. Microbiol.* **2023,** 14, 1274280.
17. Manno, G.; Dalmastri, C.; Tabacchioni, S.; Vandamme, P.; Lorini, R.; Minicucci, L.; Romano, L.; Giannattasio, A.; Chiarini, L.; Bevivino, A., Epidemiology and clinical course of *Burkholderia cepacia* complex infections, particularly those caused by different *Burkholderia cenocepacia* strains, among patients attending an Italian cystic fibrosis center. *J. Clin. Microbiol.* **2004,** 42, 1491-1497.

18. Savi, D.; De Biase, R. V.; Amaddeo, A.; Anile, M.; Venuta, F.; Ruberto, F.; Simmonds, N.; Cimino, G.; Quattrucci, S., *Burkholderia pyrrocinia* in cystic fibrosis lung transplantation: a case report. *Transplant. Proc.* **2014**, 46, 295-297.
19. Martina, P.; Bettiol, M.; Vescina, C.; Montanaro, P.; Mannino, M. C.; Prieto, C. I.; Vay, C.; Naumann, D.; Schmitt, J.; Yantorno, O., Genetic diversity of *Burkholderia contaminans* isolates from cystic fibrosis patients in Argentina. *J. Clin. Microbiol.* **2013,** 51, 339-344.
20. Coutinho, C. P.; Barreto, C.; Pereira, L.; Lito, L.; Melo Cristino, J.; Sa-Correia, I., Incidence of *Burkholderia contaminans* at a cystic fibrosis centre with an unusually high representation of *Burkholderia cepacia* during 15 years of epidemiological surveillance. *J. Med. Microbiol.* **2015,** 64, 927-935.
21. Nunvar, J.; Kalferstova, L.; Bloodworth, R. A.; Kolar, M.; Degrossi, J.; Lubovich, S.; Cardona, S. T.; Drevinek, P., Understanding the pathogenicity of *Burkholderia contaminans*, an emerging pathogen in cystic fibrosis. *PloS One* **2016,** 11, e0160975.
22. Moehring, R. W.; Lewis, S. S.; Isaacs, P. J.; Schell, W. A.; Thomann, W. R.; Althaus, M. M.; Hazen, K. C.; Dicks, K. V.; LiPuma, J. J.; Chen, L. F., Outbreak of bacteremia due to *Burkholderia contaminans* linked to intravenous fentanyl from an institutional compounding pharmacy. *JAMA Intern. Med.* **2014,** 174, 606-612.
23. Leong, L. E.; Lagana, D.; Carter, G. P.; Wang, Q.; Smith, K.; Stinear, T. P.; Shaw, D.; Sintchenko, V.; Wesselingh, S. L.; Bastian, I., *Burkholderia lata* infections from intrinsically contaminated chlorhexidine mouthwash, Australia, 2016. *Emerging Infect. Dis.* **2018,** 24, 2109.
24. Peeters, C.; Zlosnik, J. E.; Spilker, T.; Hird, T. J.; LiPuma, J. J.; Vandamme, P., *Burkholderia pseudomultivorans* sp. nov., a novel *Burkholderia cepacia* complex species from human respiratory samples and the rhizosphere. *Syst. Appl. Microbiol.* **2013,** 36, 483-489.
25. Fujii, Y.; Suwa, A.; Tsuyuki, Y.; Koyama, K.; Nio-Kobayashi, J.; Yoshii, K., The first case of a cat infected with *Burkholderia pseudomultivorans*, a member of the *Burkholderia cepacia* complex. *Vet. Sci.* **2024,** 11, 559.
26. Morales-Ruíz, L.-M.; Rodríguez-Cisneros, M.; Kerber-Díaz, J.-C.; Rojas-Rojas, F.-U.; Ibarra, J. A.; Estrada-de Los Santos, P., *Burkholderia orbicola* sp. nov., a novel species within the *Burkholderia cepacia* complex. *Arch. Microbiol.* **2022,** 204, 178.
27. Velez, L. S.; Aburjaile, F. F.; Farias, A. R.; Baia, A. D.; Oliveira, W. J.; Silva, A. M.; Benko-Iseppon, A. M.; Azevedo, V.; Brenig, B.; Ham, J. H., *Burkholderia semiarida* sp. nov. and *Burkholderia sola* sp. nov., two novel *B. cepacia* complex species causing onion sour skin. *Syst. Appl. Microbiol.* **2023,** 46, 126415.
28. Wiersinga, W. J.; Van der Poll, T.; White, N. J.; Day, N. P.; Peacock, S. J., Melioidosis: insights into the pathogenicity of *Burkholderia pseudomallei*. *Nat. Rev. Microbiol.* **2006,** 4, 272-282.
29. Dance, D. A., Ecology of *Burkholderia pseudomallei* and the interactions between environmental *Burkholderia* spp. and human–animal hosts. *Acta Trop.* **2000,** 74, 159-168.
30. Lazar Adler, N. R.; Govan, B.; Cullinane, M.; Harper, M.; Adler, B.; Boyce, J. D., The molecular and cellular basis of pathogenesis in melioidosis: how does *Burkholderia pseudomallei* cause disease? *FEMS Microbiol. Rev.* **2009,** 33, 1079-1099.
31. Kettle, A. N.; Wernery, U., Glanders and the risk for its introduction through the international movement of horses. *Equine Vet. J.* **2016,** 48, 654-658.
32. Khan, I.; Wieler, L.; Melzer, F.; Elschner, M.; Muhammad, G.; Ali, S.; Sprague, L.; Neubauer, H.; Saqib, M., Glanders in animals: a review on epidemiology, clinical presentation, diagnosis and countermeasures. *Transbound. Emerg. Dis.* **2013,** 60, 204-221.
33. Srinivasan, A.; Kraus, C. N.; DeShazer, D.; Becker, P. M.; Dick, J. D.; Spacek, L.; Bartlett, J. G.; Byrne, W. R.; Thomas, D. L., Glanders in a military research microbiologist. *New Engl. J. Med.* **2001,** 345, 256-258.
34. Brett, P. J.; DeShazer, D.; Woods, D. E., Note: *Burkholderia thailandensis* sp. nov., a *Burkholderia pseudomallei*-like species. *Int. J. Syst. Evol. Microbiol.* **1998,** 48, 317-320.

35. Lertpatanasuwan, N.; Sermsri, K.; Petkaseam, A.; Trakulsomboon, S.; Thamlikitkul, V.; Suputtamongkol, Y., Arabinose-positive *Burkholderia pseudomallei* infection in humans: case report. *Clin. Infect. Dis.* **1999,** 28.
36. Glass, M. B.; Gee, J. E.; Steigerwalt, A. G.; Cavuoti, D.; Barton, T.; Hardy, R. D.; Godoy, D.; Spratt, B. G.; Clark, T. A.; Wilkins, P. P., Pneumonia and septicemia caused by *Burkholderia thailandensis* in the United States. *J. Clin. Microbiol.* **2006,** 44, 4601-4604.
37. Glass, M. B.; Steigerwalt, A. G.; Jordan, J. G.; Wilkins, P. P.; Gee, J. E., *Burkholderia oklahomensis* sp. nov., a *Burkholderia pseudomallei*-like species formerly known as the Oklahoma strain of *Pseudomonas pseudomallei*. *Int. J. Syst. Evol. Microbiol.* **2006,** 56, 2171-2176.
38. Vandamme, P.; Peeters, C.; De Smet, B.; Price, E. P.; Sarovich, D. S.; Henry, D. A.; Hird, T. J.; Zlosnik, J. E.; Mayo, M.; Warner, J., Comparative genomics of *Burkholderia singularis* sp. nov., a low G+ C content, free-living bacterium that defies taxonomic dissection of the genus *Burkholderia*. *Front. Microbiol.* **2017,** 8, 1679.
39. Ham, J. H.; Melanson, R. A.; Rush, M. C., *Burkholderia glumae*: next major pathogen of rice? *Mol. Plant Pathol.* **2011,** 12, 329-339.
40. Jeong, Y.; Kim, J.; Kim, S.; Kang, Y.; Nagamatsu, T.; Hwang, I., Toxoflavin produced by *Burkholderia glumae* causing rice grain rot is responsible for inducing bacterial wilt in many field crops. *Plant Dis.* **2003,** 87, 890-895.
41. Weinberg, J. B.; Alexander, B. D.; Majure, J. M.; Williams, L. W.; Kim, J. Y.; Vandamme, P.; LiPuma, J. J., *Burkholderia glumae* infection in an infant with chronic granulomatous disease. *J. Clin. Microbiol.* **2007,** 45, 662-665.
42. Ura, H.; Furuya, N.; Iiyama, K.; Hidaka, M.; Tsuchiya, K.; Matsuyama, N., *Burkholderia gladioli* associated with symptoms of bacterial grain rot and leaf-sheath browning of rice plants. *J. Gen. Plant Pathol.* **2006,** 72, 98-103.
43. Nandakumar, R.; Shahjahan, A.; Yuan, X.; Dickstein, E.; Groth, D.; Clark, C.; Cartwright, R.; Rush, M., *Burkholderia glumae* and *B. gladioli* cause bacterial panicle blight in rice in the southern United States. *Plant Dis.* **2009,** 93, 896-905.
44. Graves, M.; Robin, T.; Chipman, A. M.; Wong, J.; Khashe, S.; Janda, J. M., Four additional cases of *Burkholderia gladioli* infection with microbiological correlates and review. *Clin. Infect. Dis.* **1997,** 25, 838-842.
45. Brizendine, K.; Baddley, J.; Pappas, P.; Leon, K.; Rodriguez, J., Fatal *Burkholderia gladioli* infection misidentified as Empedobacter brevis in a lung transplant recipient with cystic fibrosis. *Transpl. Infect. Dis.* **2012,** 14, E13-8.
46. Rajendraprasad, S.; Creech, Z. A.; Truong, G. T. D.; Nguyen, T.; Addula, M.; Mendoza, N.; Velagapudi, M., Fatal case of *Burkholderia gladioli* pneumonia in a patient with covid-19. *Ochsner J.* **2022,** 22, 349-352.
47. Azegami, K.; Nishiyama, K.; Kato, H., Effect of iron limitation on "*Pseudomonas plantarii*" growth and tropolone and protein production. *Appl. Environ. Microbiol.* **1988,** 54, 844-847.

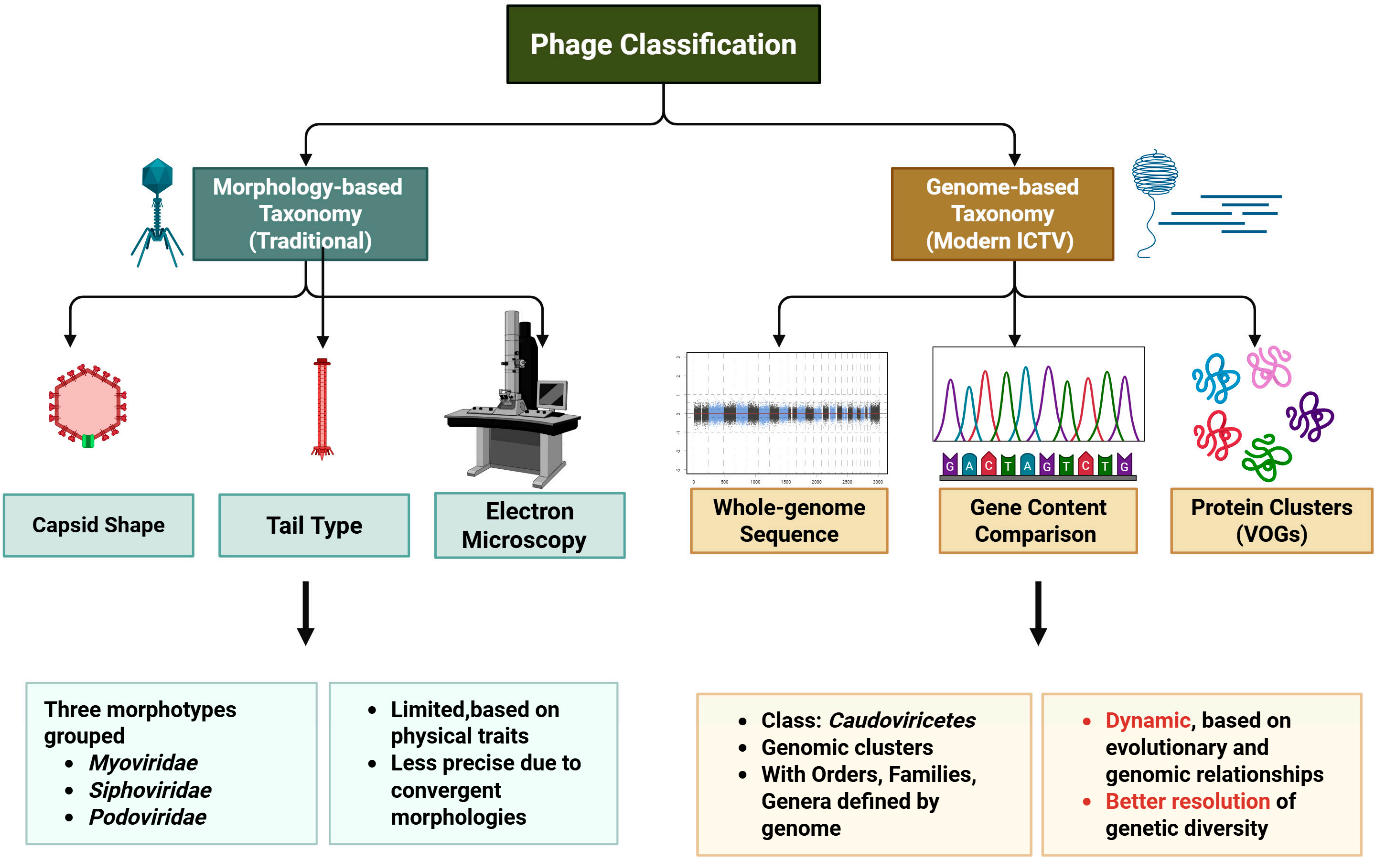

Figure S1. Comparison of morphology-based and genome-based phage classification. Conventional morphology-based phage taxonomy classifies phages by capsid shape and tail type using electron microscopy. Modern genome-based phage taxonomy classifies phages by genome sequencing, overall DNA and protein similarity, and phylogenetic analyses based on core genes and proteins. This transition improves classification in capturing phage diversity and evolutionary relationships.
